# Supplementary material for: Identification and functional analysis of non-coding regulatory small RNA FenSr3 in Bacillus amyloliquefaciens LPB-18
Source: PeerJ. 2023 May 15;11:e15236. doi: 10.7717/peerj.15236 (PMC10194069; doi:10.7717/peerj.15236)
Supplement: Supplemental Information 4 [file peerj-11-15236-s004.zip › KO/CK-vs-T1_map/map00941.html]

KEGG PATHWAY: Flavonoid biosynthesis - Reference pathway


|  |  |
| --- | --- |
| **Flavonoid biosynthesis - Reference pathway** |  |

[
Pathway menu
| Organism menu
| Pathway entry
| Show description
| User data mapping
]

Flavonoids are a major class of plant secondary metabolites that serves a multitude of functions including pigments and antioxidant activity. Flavonoids are synthesized from phenylpropanoid derivatives by condensation with malonyl-CoA. For example, condensation of p-coumaroyl-CoA (C6-C3) with three malonyl-CoA (C3) molecules results in naringenin chalcone with a diphenylpropane (C6-C3-C6) unit, which is converted to naringenin with the flavone (2-phenylchromen-4-one) backbone by conjugate ring closure. These and further modifications yield a variety of structural forms including chalcones, flavanones, dihyroflavonols, and flavans, anthocyanins, flavones and flavonols, and isoflavonoids.

|  |  |  |
| --- | --- | --- |
| Reference pathway | 184% 150% 122% 100% 82% 67% 55% | 图片下载 |
